# Portal Hypertension Unmasked—Cavernous Transformation of the Portal Vein Following Portal Vein Thrombosis in a 9‐Year‐Old Female: A Case Report

**DOI:** 10.1002/ccr3.70058

**Published:** 2025-01-06

**Authors:** Areej Motasim Khalil Seedahmed, Khalid Alshibli Dafalla Gasmalla, Hajer Yousuf Mohammed Ahmed, Sakina Eltahir Omer Mohammed, Yousif Omer Elgaili Yousif

**Affiliations:** ^1^ Pediatric Resident, Sudan Medical Specialization Board Khartoum Sudan; ^2^ Internal Medicine Resident, Sudan Medical Specialization Board Khartoum Sudan; ^3^ General Practitioner, Gezira Traumatology and Neurosurgery Specialized Centre Gezira Sudan; ^4^ Teaching Assistant at General Surgery Department Alzaiem Alazhari Unversity Khartoum Sudan

**Keywords:** cavernous transformation of the portal vein, portal hypertension, portal vein thrombosis, stress fracture, umbilical sepsis

## Abstract

This case demonstrates the complex dynamics of cavernous transformation of the portal vein and portal vein thrombus due to umbilical cord infection in a child and its consequences, namely portal hypertension. This abnormal process has to be understood for proper treatment and stresses the importance of a thorough assessment of such patients.

## 1 Introduction

Portal vein thrombosis (PVT) is defined as thrombosis that develops in the trunk of the portal vein, including its right and left intrahepatic branches. This thrombus may extend to the splenic or superior mesenteric veins [1]. The blockage of the extrahepatic portal vein (PV) with or without the participation of the intrahepatic portal branches is known as extrahepatic portal vein obstruction (EHPVO). The most common cause of EHPVO is portal vein thrombosis (PVT), while congenital anomalies such as PV stenosis, atresia, or agenesis are less common [2]. Umbilical vein catheterization (UVC) and direct injury to the portal vein, such as omphalitis, are the primary causes of extrahepatic portal vein thrombosis (EHPVT) in the pediatric age group; the incidence of PVT following umbilical vein catheterization (UVC) can approach 44% [3]. If the ductus venosus is not optimally aligned with the umbilical vein, unintentional catheterization of the left portal vein during placement may lead to severe hepatic parenchymal and vascular complications [4].

In addition, adult cases of early EHPVT following cadaveric donor liver transplantation have been reported. EHPVT following splenectomy for hematologic disorders has also been reported, albeit it is even less common in children. It is expected to find a correlation between many factors, which raises the risk of thrombosis even more. Almost half of the cases have an unidentified cause for EHPVT [5]. From infancy to adulthood, children with PVT experience portal hypertension (PH) problems at varying ages. They are referred to liver centers for a range of symptoms, such as gastrointestinal (GI) hemorrhage from variceal bleeding, splenomegaly and hypersplenism, and, less frequently, ascites. The “cavernous transformation” process of the portal vein represents an attempt to circumvent the thrombus and replace a physiological portal venous flow, or more commonly, to establish a spontaneous portosystemic shunt. Pathogenetic mechanisms leading to portal hypertension are primarily related to the increased vascular resistance in the portal venous system due to thrombus formation [2].

The present clinical case report describes a scarce and unique situation in pediatric patients and attempts to summarize the complex factors involved in the development of cavernous transformation following portal vein thrombosis secondary to umbilical vein infection. This case expands our knowledge base of this unique presentation for healthcare providers, helping them recognize and diagnose similar cases involving complex refraction in children. Additionally, this archival case study contributes to the existing medical literature by providing a detailed analysis of the sequela of events leading to portal hypertension in this specific clinical scenario, enriching the medical community's knowledge base and emphasizing the importance of vigilance in evaluating unusual presentations in pediatric patients.

## 2 Case History/Examination

A 9‐year‐old female presented with recurrent hematemesis and melena 8 h prior to admission. Further history revealed no yellowish discoloration of sclera, abdominal pain or distention, or irregular bowel habits and no bleeding from other site. There is a loss of appetite and poor weight gain since birth, as the mother mentioned. Additionally, the patient had a past medical history of a similar condition for which she went to a nearby hospital, and during her admission, she vomited blood twice, about ½ cup each, red containing clots; she was resuscitated, and on her second day of admission passed melena once. The patient had a history of repeated gastroenteritis from the age of 4 month up to 3 years where she sought medical advice several times with each time she was prescribed oral medication.

She also had past history of hematemesis twice at the age of 7 years, and 3 weeks ago the patient developed hematemesis. She sought advice missed and treated as a stress ulcer and malaria twice. There is no past history of jaundice. All other systems were normal, and the patient had a poor nutritional status, was vaccinated only with BCG, and had no family history of a similar condition. Past medical history included multiple hospitalizations and blood transfusions.

On examination, the patient appeared ill, conscious, not distressed, wasted, stunted, severe pallor, was not jaundiced, and was vitally stable. The weight was 17.5 kg below the third centile by 1 standard deviation (SD), and the height was 109 cm below the third centile by 3 SD.

Vital signs were a pulse rate of 140 beats/min, respiratory rate of 20 breaths/min, capillary refill < 2 s, and temperature was 37.5. On physical examination, abdominal examination revealed an inspection of normal contour with free flanks that move with respiration; the umbilicus was inverted, with no dilated veins, and by palpation, a soft, not tender liver three fingers below the costal margin was revealed. The liver span was 8 cm; the spleen was three fingers below the costal margin, and on percussion no shifting dullness was revealed, and on auscultation, there were normal bowel sounds.

## 3 Differential Diagnosis, Investigations and Treatment

Complete blood count (CBC) test results showed elevated WBCs with neutrophilic predominance, microcytic anemia and otherwise normal CBC as shown in Table 1 below.

**TABLE 1:** CBC results of the patient before treatment.

Lab test	Result	Reference value
WBC	13.8 × 10^3^/uL	4.5–11.0 × 10^3^/uL
Neutrophils	82.1%	40%–70%
Lymphocytes	13.2%	20%–40%
Monocytes	4.1%	2%–8%
Eosinophils	0.2%	1%–6%
Basophils	0.2%	Less than 1%
RBC	3.2 × 10^6^/uL	Male: 4.5–5.5 × 10^6^/uL Female: 4.0–5.0 × 10^6^/uL
Hb	7.3 g/dL	Male: 14–17.4 g/dL Female: 12.0–16.0 g/dL
HCT	20%	Male: 42%–52% Female: 36%–46%
MCV	64.6 fl	80–100 fl
MCH	22.4 pg	28–34 pg
MCHC	34.9 g/dL	32–36 g/dL or %
RDW_CV	19.5%	12.0%–14.6%
Platelets	182 × 10^3^/μL	150–450 × 10^3^/uL

Urine general test results showed deep yellow acidic urine with one cross sugar, three crosses acetone, and otherwise normal, as shown below in Table 2.

**TABLE 2:** Urine general results of the patient before treatment.

Test	Result	Reference value
Color	Deep yellow	Clear
pH	Acidic	5–8 pH
SG	1.005	1.020–1.050
Albumin	Nil	Negative
Sugar	+	Negative
Acetone	+++	Negative
Bile	Nil	Negative
Blood	Nil	Negative
Urobilinogen	Nil	Negative
Pus cells	2–4/hpf	0–5/hpf
RBCs	0–2/hpf	0–5/hpf

The liver function test (LFT) and coagulation profile test results revealed slightly low total protein, albumin elevated PT, APTT, INR, and otherwise normal, as shown in Table 3.

**TABLE 3:** Liver function test results of the patient before treatment.

Test	Result	Reference value
LFT		
TSP	6.1 g/dL	6.6–8.3 g/dL
S. Albumin	3.1	3.5–5.2 g/dL
S. Glob	3	1.8–3.6 g/dL
TSB	0.8	0.2–1.3 mg/dL
Direct	0.2	0.0–0.4 mg/dL
Indirect	0.6	0.0–0.9 mg/dL
Coagulation profile		
PT	20.3 s	11–13 s
PTT	44 s	26–36 s
INR	1.5	
Protein C	151%	70%–140%
Protein S	109%	70%–123%

An abdominal ultrasound was done and showed an enlarged liver of 15 cm with coarse echotexture, a portal vein measuring 23 mm filled with echogenic thrombus (PV DVT), the cavernous transformation of the portal vein, no focal lesion, a spleen enlarged in size 16 cm with normal coarse echotexture, and no ascites. An upper GI endoscopy showed multiple grade 3 esophageal varices for which they did banding. After thorough discussion, the diagnosis was portal hypertension due to portal vein thrombosis due to umbilical sepsis compensated by cavernous transformation of the portal vein. Counseling with the patient and her mother was done regarding diagnosis and treatment plan and surgical treatment also was discussing, which included trans jugular intrahepatic portosystemic shunt (TIPS) and anticoagulant.

After the banding through the upper GI endoscopy, the bleeding stopped, and the patient was put on propranolol tabs (beta‐blockers) for a regular follow‐up.

## 4 Conclusion and Results (Outcome and Follow‐Up)

On follow‐up after 1 month, she was well and not complaining of any new complaints. The monthly follow‐up was for any complications and to renew her drugs with no complications being revealed. No lab investigations were done. She was referred to a gastroenterologist for further planning.

## 5 Discussion

The case of this 9‐year‐old female patient presents a compelling clinical narrative of recurrent hematemesis and melena ultimately attributed to portal hypertension resulting from portal vein thrombosis compensated by cavernous transformation of the portal vein. The unique journey of this patient, marked by early umbilical sepsis, recurrent episodes of gastroenteritis, and subsequent misdiagnoses of severe malaria and bilharziasis, underscores the complexity and challenges in diagnosing such intricate cases, especially in the pediatric population.

One of the intriguing aspects of this case is the temporal sequence of events leading to the development of portal hypertension, including the initial insult of umbilical sepsis in early infancy, which subsequently culminated in the cavernous transformation of the portal vein and the manifestation of esophageal varices, as evidenced by upper GI endoscopy. The multi‐faceted nature of this patient's medical history, characterized by poor weight gain, recurrent hospitalizations, and blood transfusions, highlights the significant burden of disease experienced by the patient and her family. Cavernous transformation of portal vein (CTPV) often manifests from 6 to 20 days following the occurrence of portal blockage. Hepatopetal (portal‐portal shunts) and hepatofugal (portal‐systemic shunts) are two types of venous collaterals. Ascites, gastrointestinal varices, portal hypertension, splenomegaly, mesenteric venous congestion and ischemia, ascending cholangitis, and biliary cirrhosis are possible sequelae of CTPV [6]. When chronic portal vein thrombosis (CPVT) develops, the portal vein pressure often rises and the liver's blood flow is reduced, which raises the risk of variceal hemorrhage, ascites, and impaired liver function. Preventing further bleeding is crucial because variceal hemorrhage poses a serious risk to life. Anticoagulant usage in the treatment of CPVT is still debatable as of right now [7]. While one research found that anticoagulation is a safe and effective main preventive measure against PVT in adults with cirrhosis [8]; another study showed that individuals with significant PVT should carefully consider thrombolytic treatment [9]. Transjugular intrahepatic portosystemic shunt (TIPS) was not widely used in the past due to the technical difficulty of the technique for treating patients with cavernous transformation of the portal vein. The TIPS approach has seen substantial improvements during the past 10 years, and its use has grown [10].

Along with adequate control of the patient's bleeding through esophageal variceal banding and propranolol management, consultation about the possibilities of repositioning the anatomy surgically, including a transjugular intrahepatic portosystemic shunt (TIPS), demonstrates the importance placed on a multi‐faceted, co‐management approach to the difficulties of managing portal hypertension in young children. Cooperation among the healthcare providers and between the patient and her mother helped share the diagnosis and management plan, reassuring and strengthening the case management.

Moreover, the attentive follow‐up and referral to a gastroenterologist underscore the long‐term commitment to monitoring potential complications and optimizing the patient's ongoing care. The resolution of symptoms and absence of new complaints during the follow‐up period reflect the successful management of the patient's condition, highlighting the importance of regular surveillance and adherence to therapeutic regimens in ensuring positive outcomes in portal hypertension secondary to portal vein thrombosis.

In conclusion, this case highlights the importance of a thorough clinical assessment, prompt interventions, and continued care for children with complex presentations of portal hypertension. The clinical course of this patient demonstrates the ingenuity and adaptability of healthcare providers in handling the diagnostic and therapeutic conundrums associated with rare complex conditions, which ultimately confer a good prognosis for patients, carefully guiding appropriate future management strategies in similar clinical situations.

Furthermore, the detailed family history of similar medical conditions needs to be included, which may inhibit a broad reassessment of underlying predisposing factors and environmental influences in addition to potential immunization gaps related to this patient's responsiveness to infectious diseases and specific morbidities associated with these illnesses. This is a case report from a single center; therefore, the findings and management described might need to be universally generalizable. Further studies involving diverse patient populations and multiple healthcare settings are warranted to validate the recommendations and outcomes observed in this case.

## Author Contributions

**Areej Motasim Khalil Seedahmed:** conceptualization, project administration, validation, visualization, writing – original draft, writing – review and editing. **Khalid Alshibli Dafalla Gasmalla:** conceptualization, project administration, validation, visualization, writing – original draft, writing – review and editing. **Hajer Yousuf Mohammed Ahmed:** conceptualization, project administration, validation, visualization, writing – original draft, writing – review and editing. **Sakina Eltahir Omer Mohammed:** conceptualization, project administration, validation, visualization, writing – original draft, writing – review and editing. **Yousif Omer Elgaili Yousif:** conceptualization, data curation, project administration, validation, visualization, writing – original draft, writing – review and editing.

## Ethics Statement

Written informed consent was obtained from the patient's parents.

## Consent

Written informed consent was obtained from the patient's parents to publish this report in accordance with the journal's patient consent policy.

## Conflicts of Interest

The authors declare no conflicts of interest.

## Data Availability

Data are available on request from the authors.
